# Biliary-duodenal anastomosis using magnetic compression following massive resection of small intestine due to strangulated ileus after living donor liver transplantation: a case report

**DOI:** 10.1186/s40792-017-0349-4

**Published:** 2017-05-25

**Authors:** Ryusuke Saito, Hiroyuki Tahara, Seiichi Shimizu, Masahiro Ohira, Kentaro Ide, Kohei Ishiyama, Tsuyoshi Kobayashi, Hideki Ohdan

**Affiliations:** Department of Gastroenterological and Transplant Surgery, Hiroshima University, 1-2-3 Kasumi, Minamiku, Hiroshima, Hiroshima 734-8551 Japan

**Keywords:** Primary sclerosing cholangitis, Living donor liver transplantation, Magnetic compression anastomosis, Choledochoduodenostomy, Short bowel syndrome

## Abstract

**Background:**

Despite the improvements of surgical techniques and postoperative management of patients with liver transplantation, biliary complications are one of the most common and important adverse events. We present a first case of choledochoduodenostomy using magnetic compression following a massive resection of the small intestine due to strangulated ileus after living donor liver transplantation.

**Case presentation:**

The 54-year-old female patient had end-stage liver disease, secondary to liver cirrhosis, due to primary sclerosing cholangitis with ulcerative colitis. Five years earlier, she had received living donor liver transplantation using a left lobe graft, with resection of the extrahepatic bile duct and Roux-en-Y anastomosis. The patient experienced sudden onset of intense abdominal pain. An emergency surgery was performed, and the diagnosis was confirmed as strangulated ileus due to twisting of the mesentery. Resection of the massive small intestine, including choledochojejunostomy, was performed. Only 70 cm of the small intestine remained. She was transferred to our hospital with an external drainage tube from the biliary cavity and jejunostomy. We initiated total parenteral nutrition, and percutaneous transhepatic biliary drainage was established to treat the cholangitis. Computed tomography revealed that the biliary duct was close to the duodenum; hence, we planned magnetic compression anastomosis of the biliary duct and the duodenum. The daughter magnet was placed in the biliary drainage tube, and the parent magnet was positioned in the bulbus duodeni using a fiberscope. Anastomosis between the left hepatic duct and the duodenum was accomplished after 25 days, and the biliary drainage stent was placed over the anastomosis to prevent re-stenosis. Contributions to the successful withdrawal of parenteral nutrition were closure of the ileostomy in the adaptive period, preservation of the ileocecal valve, internal drainage of bile, and side-to-side anastomosis.

**Conclusions:**

Choledochoduodenostomy with magnet compression could be a less invasive and safer method for treatment of biliary stricture that cannot be accessed by conventional surgery.

## Background

Primary sclerosing cholangitis (PSC) is a chronic cholestatic disease characterized by the progressive fibrosing inflammatory destruction of the intrahepatic and extrahepatic ducts, leading to liver failure [1]. PSC is the sixth most common cause of the liver transplantation in Japanese adults, following neoplastic diseases, primary biliary cirrhosis, hepatitis C virus cirrhosis, hepatitis B virus cirrhosis, and alcoholic cirrhosis [2]. The recipient’s common bile duct is certainly resected, and hepatocholangiojejunostomy is needed in the case of liver transplantation for PSC.

Historically, surgery has been the standard treatment for biliary stricture. However, surgery may be too invasive for elderly patients or patients with a poor general and nutritional condition. Repeat surgeries for biliary stricture are difficult because of the high risk of vascular complications near the anastomosis, postoperative adhesions, and inflammatory changes [3]. There is also a risk of inflammation of the anastomosis caused by foreign bodies, such as stitches or clips [4]. However, magnetic compression anastomosis (MCA) is a less invasive and safer procedure than choledochoenterostomy or choledochocholedochostomy for a biliary stricture or obstruction, with a low rate of complications and re-stenosis [5]. This procedure is also used for biliary stricture after liver transplantation or palliation of obstructive jaundice for malignancies [4, 6]. Several reports have demonstrated the superiority of internal over external biliary drainage in terms of intestinal barrier, integrity, absorption of nutrition, and liver function [7, 8].

Here, we report a first case of choledochoduodenostomy managed with magnetic compression following a massive resection of the small intestine due to strangulated ileus after living donor liver transplantation (LDLT).

## Case presentation

The patient was a 54-year-old Chinese woman with a history of PSC and ulcerative colitis (UC). She had received LDLT using a left lobe graft, with resection of the extrahepatic bile duct and Roux-en-Y anastomosis for end-stage liver disease due to PSC. Her UC was well controlled with aminosalicylates and methylprednisolone. Cholangitis had reoccurred, but she had never previously experienced ileus in the postoperative period.

**Fig. 1:**
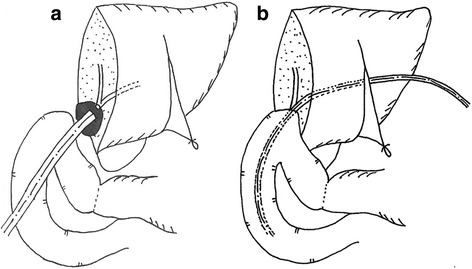
Schema of anatomical relationship between the biliary duct and duodenum **a** before magnetic anastomosis and **b** after anastomosis. **a** External drainage tube was placed in the biliary cavity and **b** PTBD tube was placed over the anastomosis

**Fig. 2:**
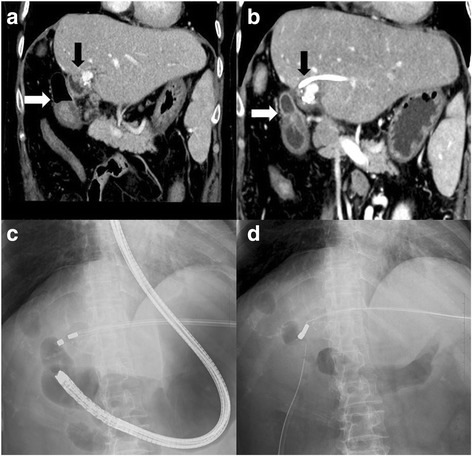
**a** Computed tomography showed the bulbus duodeni (*white arrow*) and biliary duct (*black arrow*) come in contact with each other. **b** PTBD tube was placed into B3. **c** The parent magnet was inserted from PTBD tube, and daughter magnet was placed by gastrointestinal endoscopy. **d** The parent and daughter magnets connected sandwiching the biliary wall and duodenal wall

**Fig. 3:**
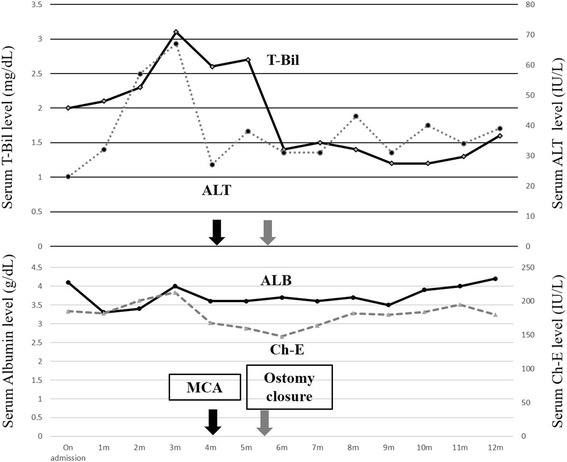
The kinetics of the hepatobiliary enzymes and nutritional indexes

**Fig. 4:**
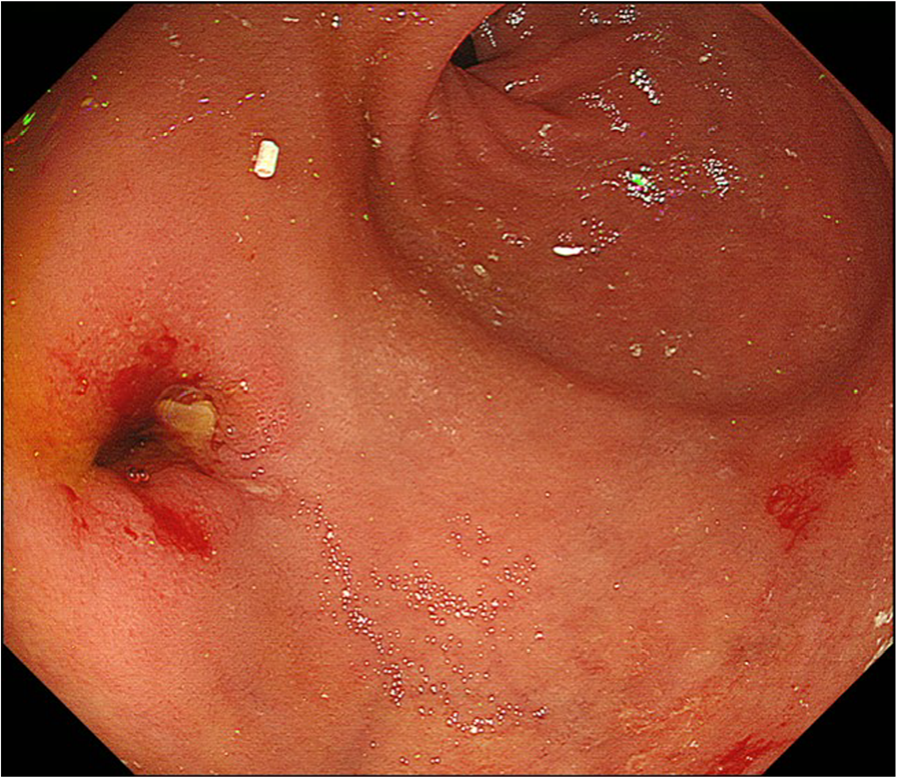
Endoscopy showed no stenosis after removal of the PTBD stent

The patient was referred to a hospital in China with sudden onset of intense abdominal pain. A strangulated ileus due to internal hernia was highly suspected, and an emergency surgery was performed. Laparotomy revealed muddy massive ascites and necrotic intestine due to twisting of the mesentery. Resection of the massive small intestine, including choledochojejunostomy, was performed, with only 70 cm of the small intestine remaining. She was transferred to our hospital with an external drainage tube of the biliary cavity and a jejunostomy raised 70 cm from the ligament of Treitz (Fig. 1a). We then initiated total parenteral nutrition for nutritional support. As a result, progressive jaundice and repeated cholangitis had occurred. The endoscopy of the biliary cavity through the external drainage tube revealed necrotic tissue and sludge. Percutaneous transhepatic biliary drainage (PTBD) to biliary duct 3 was performed to treat the cholangitis. Computed tomography showed that the bulbus duodeni and biliary duct were in close contact with a distance of 2.7 cm (Fig. 2a, b). Surgical intervention was difficult because the length of the remaining intestine was very short. Therefore, we decided to perform MCA for biliary-duodenal anastomosis. The PTBD tube was gradually replaced with thicker ones up to 16 Fr. The daughter magnet was placed in the PTBD tube, and the parent magnet was positioned in the bulbus duodeni by a fiberscope (Fig. 2c, d). Anastomosis between the left hepatic duct and the duodenum was established after 25 days, and the PTBD tube was placed over the anastomosis to prevent re-stenosis (Fig. 1b). In addition, a closure of the jejunostomy with side-to-side anastomosis was performed in the adaptive period while the capacity of the remaining intestine was at its maximum level. The results of sequential serum laboratory testing of the hepatobiliary enzymes and transition of the nutritional indexes are shown in Fig. 3. Serum levels of total bilirubin, aspartate aminotransferase, and alanine transaminase decreased after MCA and closure of the ileostomy. The body weight and serum level of albumin and choline esterase were maintained during hospitalization. The frequency of watery diarrhea decreased, and the patient was discharged 2 weeks after the closure of the ostomy without requiring parenteral nutrition. The PTBD tube was removed 11 months after the MCA (Fig. 4). After which, we looked at no stenosis of the anastomosis and improvement of hepatic function and nutritional indexes.

### Discussion

MCA is a minimally invasive procedure for the management of biliary strictures and obstruction without surgical intervention. Procedure-related complications are seldom observed, and the results of anastomotic patency are uneventful compared with conventional surgery [5]. Because MCA is an interventional procedure, it can be performed on elderly patients, those with poor general condition or who have history of multiple laparotomy. To our knowledge, this is the first report of choledochoduodenostomy with MCA after major resection of the small intestine. This is a less invasive and safer procedure for the patients who do not have enough intestine for reconstruction of the biliary duct compared with conventional surgical procedure. In our case, the preserved intestine was so short (only 70 cm including the ileocecal valve) that it would have been difficult to anastomose the biliary duct to the intestine surgically. Endoscopic ultrasonography-guided hepaticogastrostomy could be a treatment option for this case. The rate of postoperative cholangitis in hepaticogastrostomy is much higher than that of choledochoduodenostomy [9]. Retrograde cholangitis can cause potentially fatal septicemia after LDLT, so we chose choledocoduodenostomy with MCA. Maintaining both the length and condition of the remaining intestine was also an important factor for the patient’s long-term nutritional condition, allowing her to function without the need for parenteral nutrition.

Short bowel syndrome (SBS) is a disabling condition characterized by the inability to maintain fluid, electrolyte, and nutrient balances after a major resection or loss of function of the intestine [10]. Human intestine has the inherent ability to improve its absorptive capacity after a massive resection via increased intraluminal absorption and enteral neuro-hormonal stimuli, such as human growth hormone and glucagon-like peptide-2 [11, 12]. This change occurs over a period of weeks to several months after resection, known as the adaptive period [13]. External biliary drainage has a negative impact on bile acid circulation, which is important for hepatic bile formation and absorption of dietary lipids and fat-soluble vitamins in the small intestine [14]. Internal drainage of bile with MCA has positive effects on the maintenance of the nutritional condition. Closure of the ostomy during this period, preservation of the ileocecal valve, preoperative nutritional support, side-to-side anastomosis, and internal drainage of the bile all contributed to the control of watery diarrhea and recovery from malnutrition in this case.

Choledochoduodenostomy differs from the choledochoenterostomy or choledochocholedochostomy in that the magnets are used not for recanalization of the stenosis or obstruction but for creation of a new fistula. Alvalani et al. reported good results after choledochoduodenostomy with MCA for patients with a malignant obstruction [4]. The main complications after choledochoduodenostomy are sump syndrome and cholangitis. Sump syndrome occurs from bile stasis and reflux of duodenal contents into the biliary tree, resulting in cholangitis or hepatic abscess. Although the prevalence of sump syndrome is reported between 2.4 and 5% in previous studies, these cases can be managed safely and successfully with endoscopic procedure [4, 15, 16]. The rate of subsequent cholangitis after choledochoduodenostomy for benign obstruction is reported 0 to 1.1%, which is extremely lower than the rate after hepaticogastrostomy or drainage using plastic stent [9, 15, 17].

This is the first report of choledochoduodenostomy using magnetic compression after liver transplantation. In addition, there is no report of choledochoduodenostomy after major resection of the small intestine. To our knowledge, only 22 cases, including our case, of MCA after liver transplantation for biliary stricture have been reported (Table 1) [6, 18–24]. The median age of the patients was 53.5 years (range 0.5–64 years), and 13 patients were male. Liver transplantation was performed for liver cirrhosis (LC) with hepatocellular carcinoma (HCC) in nine patients, for LC with viral infection in three patients, and for fulminant hepatitis in three patients. Choledochocholedochostomy was carried out for these patients after duct-to-duct anastomosis, and choledochojejunostomy was done after duct-to-jejunum anastomosis. MCA failed in two patients: One patient developed relatively distance from the anastomosis site, and the other had diffuse narrowing and torsion of the common biliary duct, which made it impossible to place the parent magnet. Distance between the two magnets is a very important factor for successful intervention, because the strength of the magnets gets weaker as the distance gets longer [5]. Preoperative imaging study is necessary to investigate the distance between the biliary duct and intestine and also to avoid trapping the blood vessels between the two magnets. Median follow-up period reported was only 11.4 months, so further careful follow-up is necessary to determine the long-term outcome after MCA. It is reported that PSC is strongly associated with inflammatory bowel disease (IBD); in fact, its rate of incidence among patients with IBD is 75% [1]. The UC of our patient was well controlled with oral aminosalicylates and methylprednisolone. Patients with both PSC and UC have a high risk of colon cancer; therefore, careful follow-up with colonoscopy is indicated to prevent further resection of the intestine [25].

**Table 1:** Outcomes of magnetic compression anastomosis for biliary stricture after liver transplantation

Author [references]	Age	Gender	Disease	Operation	Type of intervention	Duration from LT (months)	Period until removal of magnets (days)	Duration of internal catheter (weeks)	Follow-up period (months)	Reintervention
Mimuro et al. [18]	1	F	Fulminant hepatitis	LDLT of left lobe with R-Y	CJS	6	12	8	54	None
	57	M	LC with HCC	LDLT of right lobe with R-Y	CJS	7	12	8	6	Balloon dilatation
Okajima et al. [6]	44	F	Fulminant hepatitis	LDLT of right lobe with D-D	CCS	12	42	12	15	None
Akita et al. [19]	34	F	N/A	LDLT of right lobe with D-D	CCS	0	21	None	N/A	N/A
Mita et al. [20]	0.5	F	Fulminant hepatitis	LDLT of lateral segment with R-Y	CJS	1.5	12	N/A	73.4	None
	59	F	LC	LDLT of left lobe with R-Y	CJS	5	25	N/A	62.2	ESWL
Matsuno et al. [21]	53	M	N/A	LDLT of right lobe with D-D	CCS	N/A	10	4.3	36	None
Itoi et al. [22]	60	M	N/A	LDLT of right lobe with D-D	CCS	18	9	24	2	None
Jang et al. [23]	63	F	LC with HCC (HBV)	LDLT with D-D	CCS	71	42	83	19.4	None
	49	M	LC with HCC (HBV)	LDLT with D-D	CCS	6	26	70	16.4	None
	54	M	LC with HCC (HBV)	LDLT with D-D	CCS	55	18	49	11.4	None
	64	F	LC with HCC (HBV)	LDLT with D-D	CCS	71	71	14	3.3	Balloon dilatation
	54	M	LC (HBV)	LDLT with D-D	CCS	15	102	26.7	9.5	None
	48	M	LC with HCC (HBV)	LDLT with D-D	CCS	13	102	25.7	8.8	None
	63	F	HF (drug-induced)	LDLT with D-D	Failure	107	–	–	–	–
	33	M	HF (HAV)	LDLT with D-D	CCS	5	33	26.7	10.4	None
	61	M	LC with HCC (HBV)	LDLT with D-D	CCS	4	14	25.4	96.9	None
	54	M	LC with HCC (HBV)	LDLT with D-D	CCS	9	181	7.3	4.9	None
	52	M	LC (HBV)	LDLT with D-D	CCS	1	153	indwelling	N/A	None
	51	M	LC (HBV)	LDLT with D-D	Failure	5	–	–	–	–
Perez-Miranda et al. [24]	53	M	N/A	Orthotopic LT with D-D	CCS	36	10	Indwelling	N/A	None
Present case	54	F	LC (PSC)	LDLT of left lobe with R-Y	CDS	60	25	44	4	None

*CCS* choledochocholedochostomy, *CDS* choledochoduodenostomy, *CJS* choledochojejunostomy, *ESWL* extracorporeal shock wave lithotripsy, *D-D* duct-to-duct anastomosis, *F* female, *HAV* hepatitis type A virus, *HBV* hepatitis type B virus, *HCC* hepatocellular carcinoma, *HF* hepatic failure, *LC* liver cirrhosis, *LDLT* living donor liver transplantation, *LT* liver transplantation, *M* male, *N/A* not addressed, *PSC* primary sclerosing cholangitis, *R-Y* Roux-en Y anastomosis

## Conclusions

Biliary-duodenal anastomosis could be an alternative method for the management of biliary stricture or obstruction. In addition, MCA could be a less invasive method for treatment of biliary stricture that cannot be accessed by conventional surgery. Careful follow-up of these cases should be done to determine the long-term patency and complications in these patients.

## Abbreviations

LDLT: Living donor liver transplantation
MCA: Magnetic compression anastomosis
PSC: Primary sclerosing cholangitis
PTBD: Percutaneous transhepatic biliary drainage
UC: Ulcerative colitis
